# Nest Architecture, Colony Productivity, and Duration of Immature Stages in a Social Wasp, *Mischocyttarus consimilis*

**DOI:** 10.1673/031.010.19101

**Published:** 2010-11-03

**Authors:** Thiago S. Montagna, Viviana O. Torres, Wedson D. Fernandes, William F. Antonialli-Junior

**Affiliations:** ^1^ Centro Integrado de Anáálise e Monitoramento Ambiental, Universidade Estadual de Mato Grosso do Sul, Dourados - MS, Brazil; ^2^ Faculdade de Ciêências Biolóógicas e Ambientais, Universidade Federal da Grande Dourados, Dourados - MS, Brazil

**Keywords:** independent foundation, Mischocyttarini, Neotropical wasp, Polistinae

## Abstract

This study examined the nest architecture, colony productivity, and duration of the immature stages of the social wasp *Mischocyttarus consimilis* Zikáán (Hymenoptera: Vespidae). The study was carried out under field conditions. Nests of *M. consimilis* consist of a single uncovered comb, which is attached to the substratum by a single petiole. The data for the nest architecture showed a positive and significant correlation between the size of the comb and the diameter of the petiole, and also between the height and diameter of the cells. The nests were constructed on horizontal, vertical, and sloping substrata with no apparent preference for a specific orientation. The colonies produced 72.9 cells and 40.7 adults on average. The mean frequency of productive cells was 33.3%, and 19.4% of the cells were reused. The mean duration of the immature stages combined was 69.7 days and the egg, larval, and pupal stages had mean durations of 14.9, 36.0, and 18.8 days, respectively. The duration of each immature stage was significantly shorter in the warmhumid season, and the larval and pupal stages were shorter during the colony pre-emergence stage.

## Introduction

The construction of exposed nests is one of the principal characteristics of social life for most polistine wasps (Starr 1991). To construct their nests, these wasps generally use plant fiber, which after being mixed with water and probably a salivary secretion gives a paper-like final product. The paper or carton may vary considerably in thickness and texture among the several groups of wasps (Richards and Richards 1951). In polistines, the nests are also variable in their architectural arrangement mainly with regard to the presence or absence of the covering envelope and combs with or without a petiole (Wenzel 1991, 1998). These variations have considerable taxonomic value and have contributed substantially to the classification of this group (Richards 1978; Wenzel 1991, 1998). Hunt and Carpenter (2004) noted that, besides its taxonomic value, nest architecture in social wasps can be used to address phylogenetic, behavioral, ecological, and evolutionary questions. In more-derived groups of Polistinae such as *Polybia,* the nest is formed by several combs covered by an envelope, and is attached to the substratum without a petiole (Jeanne 1975; Wenzel 1998). In contrast, in less-derived groups such as *Mischochyttarus,* the nest is formed by a single uncovered comb, which is attached to the substratum by a petiole (Jeanne 1975; Wenzel 1998). With few exceptions, most of the wasps that do not close their combs with an envelope suspend the nest by means of a narrow petiole, and most of those that build fixed cells directly on the substratum enclose the nest with a protective envelope (Jeanne 1975).

In *Mischochyttarus,* the colony foundation may be either haplometrotic or pleometrotic (Gadagkar 1991). Several studies have suggested that there is a close relationship between the pattern of foundation and the success of the colony. In general, the success of a colony is measured through the investment in individual production and the colony survival (Lorenzi and Turillazzi 1986; Reeve 1991). Gamboa (1978) observed that colonies of *Polistes metricus* founded by association are significantly more productive than colonies initiated by a single female. Similarly, Gamboa et al. (1992) and Tibbetts and Reeve (2003) observed for *P. fuscatus* and *P. dominulus,* respectively, that colonies initiated by association are better able to defend against predators and conspecific wasps. Environmental factors have also been linked to the success of the colonies. Inagawa et al. (2001) and Nadeau and Stamp (2003), for example, showed that colony productivity in *P. snelleni* and *P. fuscatus,* respectively, was higher for colonies located in warmer situations. Because of the importance of these aspects for the evolution and maintenance of social behavior in wasps, the objective of this study was to describe the nest architecture, colony productivity, and the duration of the immature stages in *Mischocyttarus consimilis* Zikáán (Hymenoptera: Vespidae).

## Methods and Materials

### Study location and climate characterization

The study was carried out in an area of approximately 20,000 m^2^ on the campus of the Universidade Federal da Grande Dourados, in the municipal district of Dourados (latitude 22°° 13′? 16″? S; longitude: 54°° 48′? 20″? W), state of Mato Grosso do Sul in central-western Brazil. The climate, according to the classification of Zavatini (1992), is humid subtropical with more precipitation and higher temperatures in September through February (warm-rainy season), and less precipitation and milder temperatures in March through August (cold-dry season).

### Data collection

Data were collected for a total of 33 colonies from May 2007 through June 2008. The colonies were observed under field conditions. For the study of nest architecture, the following variables were measured (n = 8 nests): length and diameter of the petiole, length and width of the nest, and length and width of the cells. For the study of productivity, the following parameters were analyzed (n = 14 nests): duration of the colonies, in days; number of cells constructed; number of adults produced; maximum number of generations in the most productive cells of the comb; and number of productive cells (cells that produced adults). The duration of the immature stage was measured from the total durations of the egg, larva, and pupa stages for each season. Those individuals in which the development continued through the two seasons were discarded from the sample. For the egg stage, only data for those eggs that remained in the same position in the cell from egg-laying to larval eclosion were used.

Most of the colonies were observed from their foundation until they were abandoned. In order to find possible foundations, the study area was monitored weekly. Data were collected on established colonies three times a week on designated days (during the collection of data, drawings of each nest were made, followed by a description of the contents of each cell of the comb). By this method, an accurate report of the productivity of each colony was obtained. In cases where it was not possible to identify the exact date of foundation, the information on adults' productivity was inferred from the number of meconia layers. The meconia layers were counted from a longitudinal section of each cell of the comb.

**Table 1.**
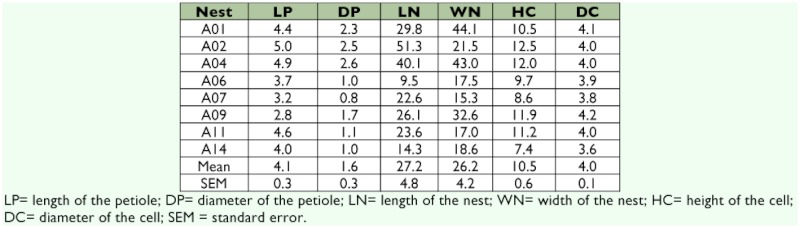
Morphometric data (mm) for eight post-emergent nests of *Mischocyttarus consimilis*

### Statistical analysis

Correlation analyses were performed to determine the relationship between the variables of each nest, as well as between the variables of colony productivity. The *t*-test for two independent samples was used to examine for differences in the duration of the immature stage through the seasons and the stages of the colony. A level of significance of 0.05 was applied throughout.

## Results

### Nest architecture

The nests of *M. consimilis* are composed of a single, uncovered comb which is attached to the substratum by a single petiole that is invariably positioned in the central area of the comb and forms an angle of 90°° to the comb. For the eight nests analyzed (Table 1), the mean length and diameter of the petioles were 4.1 ±± 0.3 and 1.6 ±± 0.3 mm, respectively (mean ±± SEM). The mean length and width of the combs were 27.2 ±± 4.8 and 26.2 ±± 4.2 mm, respectively. Post-emergence nests located in areas less affected by human activities tended to have combs that were larger than the mean size (personal observation). The final arrangement of the comb for nests in completely open locations was usually elliptical, although the arrangement was adjusted to the available space in the nesting sites. The mean height and diameter of the cells were 10.5 ±± 0.6 and 4.0 ±± 0.1 mm, respectively. The cells were added to the comb in a random way, so that it was not possible to predict the arrangement of the nest as it grew; however, the cells were added preferentially in areas of the comb where less material seemed to be needed. The cells added to the comb were round in outline and became hexagonal when they were surrounded by other cells.

**Figure 1.**
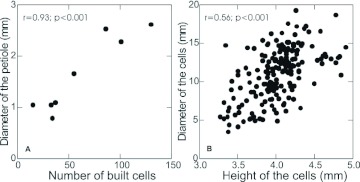
Correlation analyses for post-emergence nests of *Mischocyttarus consimilis.* A) diameter of petiole and number of cells constructed; B) Diameter and height of cells. High quality figures are available online.

There was a significant positive correlation between the diameter of the petiole and the number of cells in the comb (r=0.93; *p*<0.01; n=8) (Figure 1A). The petiole was enlarged by the addition of wood pulp. The cells became progressively larger in diameter in the distal direction, with a significant positive correlation between diameter and height (r=0.56; *p*<0.01; n=191) (Figure 1B). For each emerged adult, a new meconia layer was deposited on the bottom of the used cell. The central cells in abandoned nests were relatively larger than cells on the periphery.

**Figure 2.**
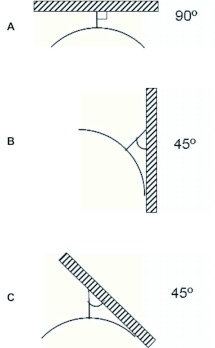
Nest-building patterns of colonies of *Mischocyttarus consimilis* according to the orientation of the substratum and the angle formed between the substratum and petiole. A) horizontal substratum; B) vertical substratum; C) sloping substratum. High quality figures are available online.

Horizontal, vertical, and sloping substrata were used by *M. consimilis* to affix the nest (Figure 2), and there was no apparent preference for any orientation. The smaller angle between the substratum and the petiole ranged from 45°° to 90°°. On horizontal substrata this angle was approximately 90°° (Figure 2A), whereas on vertical and sloping substrata it was approximately 45°° (Figures 2B and 2C).

### Colony productivity

The mean duration of the colonies of *M. consimilis* was 231.7 ±± 25.3 days (Mean ±± SEM) (Table 2). Colonies that reached the post-emergence phase produced a mean of 72.9 ±± 10.6 cells (Table 2); the smallest and largest colonies produced 37 and 159 cells, respectively (Table 2). Colonies produced a mean of 40.7 ±± 14.0 adults (Table 2). The frequency of productive cells was 33.3 ±± 4.9%, and of reused cells was 19.4 ±± 4.9%. The maximum numbers of generations in the most productive cells averaged 2.7 ±± 0.4 (Table 2). For every two cells constructed approximately one adult emerged, and these colonies produced an adult every six days and a cell every three days on average.

The correlation analysis demonstrated that the longevity of the colonies affected the number of adults produced (r=0.63; *p*=0.03; n=11), the number of cells constructed (r=0.85; *p*<0.01; n=11), and the number of productive cells (r=0.69; p=0.02; n=11) (Table 3). The size of the nests was associated with the number of adults produced (r=0.90; *p*<0.01; n=14), the number of productive cells (r=0.93; *p*<0.01; n=14), and the number of reused cells (r=0.82; *p*<0.01; n=14). The number of adults produced was associated with the number of productive cells (r=0.98; *p*<0.01; n=14) and the number of reused cells (r=0.96; *p*<0.01; n=14). An association was also found between the number of productive cells and the number of reutilizations of cells (r=0.91; *p*<0.01; n=14).

### Duration of the immature stages

The durations of the egg, larval, and pupal stages were 14.9 ±± 0.3; 36.0 ±± 0.8, and 18.8 ±± 0.4 days, respectively (Mean ±± SEM) (Table 4). Therefore the mean development time from when the egg was laid to the emergence of the adult was 69.7 days (Table 4). The mean duration of each immature stage was significantly longer during the cold-dry season than the warm-rainy season (Table 5). Comparing the two phases of the colony cycle, the duration of the larval and pupal stages was significantly shorter for colonies in pre-emergence than for colonies in postemergence (Table 6).

## Discussion

### Nest architecture

The architecture of the nests of *M. consimilis* is similar to that described for other groups with independent foundation within the Polistinae, and is classified as ““gymnodomous-stelocyttarous”” (Jeanne 1975). The characteristic central insert of the petiole on the comb in *M. consimilis* differs from other, similar species in the same study area, for instance *M. cerberus,* which has an outlying petiole (Giannotti 1999). Seemingly, in *M. consimilis* the location for the colony was selected independently of the orientation of the substratum. This pattern is different from *P. biglumis,* which prefers to place its colonies on vertical substrata, and *P. snelleni* which nests exclusively on horizontal substrata (Yamane 1969).

The relationship between the diameter of the petiole and the number of cells in the comb was similar to the results reported by Downing and Jeanne (1986) for *P. fuscatus* and *P. instabilis.* In those groups, the increase in petiole diameter is partly associated with the application of wood pulp (Downing and Jeanne 1986). These authors stated that enlargement of the petiole by wood pulp only occurs in independent-foundation species in temperate regions. However, this phenomenon was observed in our study, suggesting that the capacity to add wood pulp to the petiole can also be a characteristic of tropical species. It is possibly related, in species of both climate areas, to the need to stabilize the comb as the colony grows. The relationship between the height and diameter of the cell suggests that in this species, the cells enlarge in response to the development of the larva and the number of times that the cell is used. For each emerged adult, a new meconia layer was deposited on the bottom of the used cell. In addition, we observed that the central cells in abandoned nests were larger than cells of the periphery. Similar observations were reported by Downing and Jeanne (1986) for eight species of *Polistes,* and by Yamane (1972) for declining nests of *P.* *chinensis.*

**Table 2.**
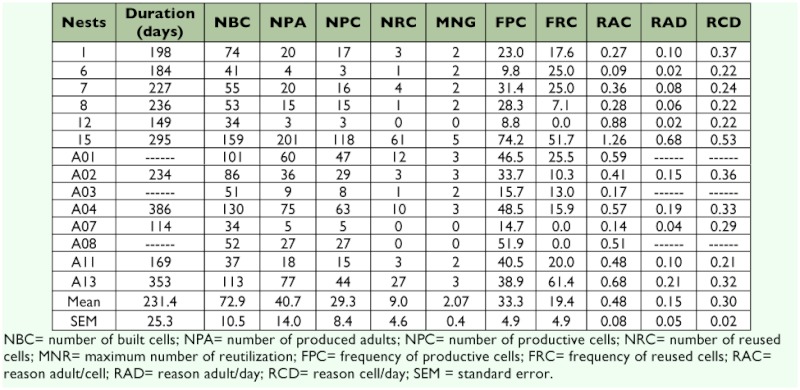
Productivity of 14 colonies of *Mischocyttarus consimilis,* observed from their foundation through decline.

**Table 3.**
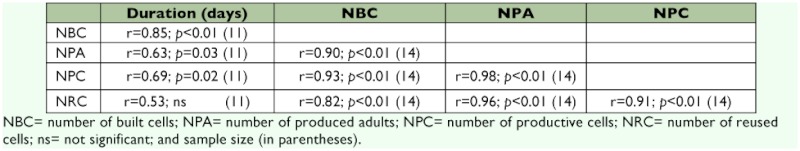
Correlation analyses between the variables of colony productivity in *Mischocyttarus consimilis.*

**Table 4.**
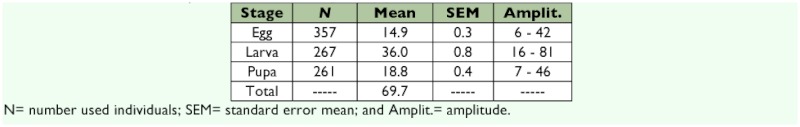
Duration of development of immature stages (days) in *Mischocyttarus consimilis*

**Table 5.**
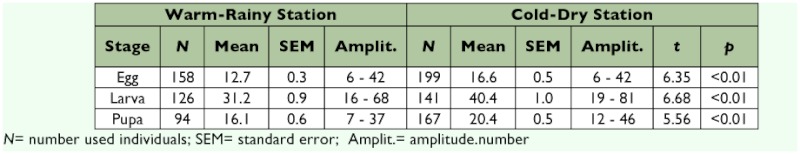
Duration of development of immature stages (days) in *Mischocyttarus consimilis,* during the warm-rainy and cold-dry seasons, and the values of the *t*-test between the two seasons.

**Table 6.**
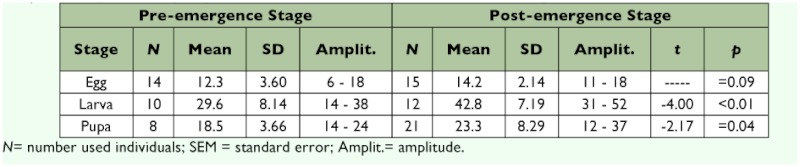
Duration of development of immature stages (days) in *Mischocyttarus consimilis* colonies during the pre-emergence and post-emergence colony stages, and the values of the *t*-test between the two stages.

**Figure 3.**
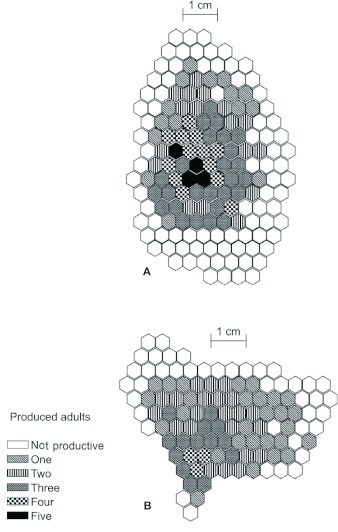
Productive and reused cells in two nests of *Mischocyttarus consimilis,* monitored from foundation to abandonment: (A) colony 15; (B) colony 22. High quality figures are available online.

### Colony productivity

In colonies of *M. consimilis,* cells continued to be added and adults produced throughout the year. This constant production is a result of the asynchrony in the colony cycle of tropical species, in which colonies in different phases occur simultaneously in the population in every season of the year. The results of this study demonstrated that longer-lived colonies, independently of the season, produced more cells and therefore grew larger consequently containing more productive and reutilized cells (Table 2). These colonies of *M. consimilis* had a longer mean duration than those described by Rocha et al. (2007) for *M. cearensis* and by Penna et al. (2007a) for *M. drewseni.* However, the numbers of cells constructed and adults produced were similar to the observations of Penna et al. (2007b) for the productivity of colonies of *M. cassununga,* although lower than the values estimated by Penna et al. (2007a) for the mean productivity in *M. drewseni.*

Although new cells were constructed continuously during the entire colony cycle, the great majority were unproductive (Table 2). However, many unproductive cells were not necessarily always empty; in other words, in most there were attempts to rear offspring. Although this aspect was not investigated in detail, we observed that the incidence of abortion was lower in the more-central cells in the comb (Figure 3). Possibly the offspring that grow in the central area of the comb receive more parental care, since the adults monitor this area of the nest more frequently (Giannotti and Machado 1999). In addition, the age of the colony did not affect the mean number of reused cells (Table 2). This observation concords with the description of Penna et al. (2007b) for both *M. cerberus* and *M. cassununga.*

### Duration of the immature stages

The duration of the egg stage was similar to the values reported for other species of the same genus. Litte (1979) estimated a mean duration of 14.1 days for *M. flavitarsis,* and Giannotti and Fieri (1991) reported a mean duration of 13.2 days for *M. cassununga.* The duration of the larval stage in *M. consimilis* was longer than those reported for other congeners. Jeanne (1972) reported a mean duration of 20.2 days for *M. drewseni,* Litte (1977) estimated 24.8 days for *M. mexicanus,* and Silva and Oliveira (1989) estimated 25.1 days for larvae of *M. atramentarius.* The duration of the pupal stage was similar to those described for other species of the genus. Litte (1979) described a mean duration of 19.7 days for pupae of *M. flavitarsis,* and later (Litte 1981) estimated a mean duration of 16.3 days for pupae of *M. labiatus.*

Giannotti (1997) suggested that among other factors, the development of the immature stage can be affected by environmental factors, food, foraging efficiency, predators, and parasites. In this study, it was observed that the development time of the offspring is longer in the cold-dry season than in the warm-rainy season, suggesting a direct effect of environmental variables on the development of the immature stage. Among the environmental factors that influence the development of immature individuals, temperature has received much attention (Jeanne 1972). Studies on the effect of temperature on the development of insects indicate that high temperatures accelerate their metabolic processes and shorten the development time (Howe 1967; Gilbert and Raworth 1996; Whitfield and Richards 1992). Thus, our results confirm the effect of negative changes of temperature on the duration of the immature stage in social wasps. Additionally, Jeanne (1972), Mead et al. (1994), and O' Donnell and Joyce (2001) stated that the duration of the larval stage, as opposed to the other immature stages, is affected by the amount of food received in this stage. Thus, a poorly fed larva tends to have a longer development time than a wellfed larva. Larvae that do not receive enough food to allow pupation must usually remain in the cell for a longer period. In this way, larval development in social wasps may be indirectly affected by the temperature, because in low temperatures a colony will forage less for food (Giannotti 1997).

In *M. consimilis,* the larval stage is shorter in pre-emergence colonies. Clouse (2001) reported that for *M. mexicanus* the larval development is shorter in pre-emergence colonies initiated by small groups, and the duration increases significantly with the number of foundresses. It is probable that a larger number of adults would increase the per-capita foraging capacity. These data suggest that the adults can manipulate larval development because the larvae are completely dependent on them for food. On this subject, Gamboa (1980) demonstrated that the larval development accelerated in *P. metricus* during the pre-emergence colony stage, but no evidence was found for a synergistic action of the number of adults on this acceleration. Other approaches suggest that larval development in pre-emergence colonies in primitively eusocial wasps may be influenced indirectly by the incidence of predation at the nest sites. As a way of increasing the number of adults and consequently the defense potential of the colony, the foundress may accelerate the development of the first larvae (Gamboa 1980). Gamboa et al. (1992) and Itôô and Itioka (2008) showed that the susceptibility of initial colonies in independent-founding species to the attack of predators or conspecific wasps is closely associated with the number of adults in the colony. That is, colonies that are initiated by fewer foundresses are more susceptible to attack by predators and must reduce the time to produce the first workers (Gamboa 1978; Itôô and Itioka 2008; Clouse 2001).
